# Fractal Characteristics of Soil Retention Curve and Particle Size Distribution with Different Vegetation Types in Mountain Areas of Northern China

**DOI:** 10.3390/ijerph121214978

**Published:** 2015-12-03

**Authors:** Xiang Niu, Peng Gao, Bing Wang, Yu Liu

**Affiliations:** 1 State Key Laboratory of Forest Ecology and Environment, China’s State Forestry Administration, The Research Institute of Forest Ecology, Environment and Protection, Chinese Academy of Forestry, Beijing 100091, China; Niuxiang@caf.ac.cn (X.N.); wangbing@caf.ac.cn (B.W.); 2 College of Forestry/Mountain Tai Forest Ecosystem Research Station of State Forestry Administration/Shandong Provincial Key Laboratory of Soil Erosion and Ecological Restoration, Shandong Agricultural University, Tai’an 271018, China; smile123liuyu@163.com

**Keywords:** soil particle, fractal method, monofractal and multifractal dimension, Return Farmland to Forests Project

## Abstract

Based on fractal theory, the fractal characteristics of soil particle size distribution (PSD) and soil water retention curve (WRC) under the five vegetation types were studied in the mountainous land of Northern China. Results showed that: (1) the fractal parameters of soil PSD and soil WRC varied greatly under each different vegetation type, with *Quercus acutissima* Carr. and *Robina pseudoacacia* Linn. mixed plantation (QRM) > *Pinus thunbergii* Parl. and *Pistacia chinensis* Bunge mixed plantation (PPM) > *Pinus thunbergii* Parl. (PTP) > *Juglans rigia* Linn. (JRL) > abandoned grassland (ABG); (2) the soil fractal dimensions of woodlands (QRM, PPM, PTP and JRL) were significantly higher than that in ABG, and mixed forests (QRM and PPM) were higher than that in pure forests (PTP and JRL); (3) the fractal dimension of soil was positively correlated with the silt and clay content but negatively correlated with the sand content; and (4) the fractal dimension of soil PSD was positively correlated with the soil WRC. These indicated that the fractal parameters of soil PSD and soil WRC could act as quantitative indices to reflect the physical properties of the soil, and could be used to describe the influences of the Return Farmland to Forests Projects on soil structure.

## 1. Introduction

The soil particles-size distribution (PSD) and the soil water retention curve (WRC) are important soil physical properties due to their influence on soil hydraulic characteristics, fertility and erosion [1,2]. Describing the soil PSD and the soil WRC quantitatively are important for soil structure research [3]. Using the monofractal and multifractal dimension to describe soil structure, dynamics, and physical processes are becoming more popular because it provides a better understanding of soil system performance. In particular, more and more people pay attention to describing soil PSD and WRC by the fractal theory and method [4].

Since Tyler and Wheatcraft [5] proposed the fractal dimension calculation formula for the soil PSD, this fractal theory has been widely used in soil research. Using Turcotte’s [6] model, Bittelli *et al.* [7] found that three domains characterized the cumulative PSD of 19 soils, and they correlated the power exponent in each domain with the fractal dimensions of the soil’s clay, silt, or sand content. Using a monofractal analysis, Wang *et al.* [8] studied the soil fractal features to predict the fractal dimensions of soil structure and the soil properties of typical forest stands of Jinyun Mountain in Chongqing, China, and they also correlated the fractal features with the changes in soil properties. By studying the monofractal characterization of soil PSD under different forest stands in China’s Dahei Mountain, Gao *et al.* [9] found that the fractal dimension of soil particles was significantly higher in woodlands than that in grasslands, higher in natural forests than that in artificial forests and greatest in natural secondary mixed forests. Using monofractal analysis, Tripathi *et al.* [10] researched the distribution characteristics of soil aggregates from the Indian tropical ecosystem. From these soil fractal studies, it became evident that models based on a monofractal dimension cannot adequately describe soil particle and pore size distributions [11,12].

In recent years, different soil PSD data sets have been characterized by their multifractal spectra [13]. Using the multifractal method, Wang *et al.* [14] analyzed the fractal characteristics of soil PSDs of different land types in the hilly and gully regions of the Loess Plateau in China. Moreover, some scholars applied the fractal theory to investigate the soil WRC and used the fractal dimensions of the soil WRC to describe the corresponding soil WRC [15,16]. Especially, Ghanbarian and Hunt [16] reviewed three well-known fractal approaches, Kravchenko and Zhang, the pore-solid fractal, and Hunt and Gee [17] methods, which estimated WRC from particle size distribution. They argued that the most reliable method was to use Hunt and Gee’s approach to estimate WRC, then modified the estimated equilibrium water content by the Hunt and Skinner algorithm for non-equilibrium conditions [17]. However, researches on the fractal dimensions of the soil WRC are still limited, and our understanding of the relationship between the soil PSD and WRC.

Mountainous land in southern Shandong province is typical of the rocky mountain areas of northern China, which has thin, loose, and high soil and water loss. Since the 1980s, with the implementation of ecological, environmental construction projects, such as the Return Farmland to Forests Project, many afforestation vegetation types have been developed, and there have been many reports written on ecological afforestation technologies and soil hydrological benefits [18,19]. However, there are generally few quantitative studies on the fractal characteristics of the soil structure, PSD and WRC in the mountainous area of northern China, making it difficult to assess the ecological effects of environmental construction projects (such as Return Farmland to Forests Project) on soil structure and function in the study area. Based on monofractal and multifractal theory, this study describes the fractal characteristics and correlation of the soil PSD and WRC in the five vegetation types in the mountainous land of southern Shandong province of Northern China. It can provide the theoretical basis for the construction of Return Farmland to Forests Project and the evaluation of forest ecological service function in the mountainous land of Northern China.

## 2. Materials and Methods

### 2.1. Study Area Condition

**Figure 1:**
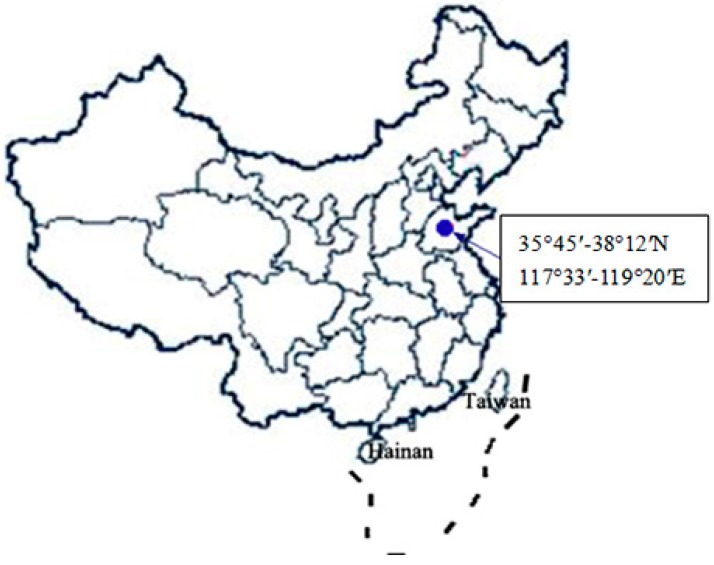
Map of the study area generated by Auto CAD2007 software. The dot on the map indicates the study area.

The experiment was conducted in Xintai City of Shandong Province (35°45′–38°12′N, 117°33′–119°20′E), located in the mountainous land of south-central Shandong Province in China (Figure 1). This area is typical monsoon climate and is located in a warm temperate zone with distinct seasonal changes. The mean annual precipitation is 798.4 mm. The average annual evaporation in this region is 1942.6 mm, and the mean annual temperature is approximately 12.0 °C. The soil type in this study is referred to as Brown soil and is similar to the American soil classification of Eutrochrepts, soil layer thickness is 15–20 cm, and is subject to high soil and water loss. Thus, strengthening the ecological environment management of the study area is very necessary. According to the floristic-vegetational analysis results [20], the vegetation types of study area belong to the coniferous forests and deciduous broad-leaved forests in the warm temperate zone, which belongs to north China flora of China. The study area includes the following major land use practices: the forest land (the coniferous forests includes *Pinus thunbergii* Parl. and *Platycladus orientalis* (L.); the deciduous broad-leaved forests includes *Quercus acutissima* Carr., *Robina Pseudoacacia* Linn., *Juglans rigia* Linn. and *Castanea mollissima* BL.; the species under the woodland vegetation are *Pistacia chinensis* Bunge, *Lespedeza bicolor* Turcz., *Spiraea salicifolia* L. and *Cotinus coggygria* Scop., *etc*.), the abandoned grassland (the species are *Zoysia japonica* Steud., *Rubia manjith* Roxb. ex Flem., *Themeda japonica* Tanaka and *Setaria viridis (Linn.)* Beauv, *etc.*), and the farmland (including *Zea mays*, peanuts and vegetables).

### 2.2. Sampling and Processing

This work was based on Forestry Standards “Observation Methodology for Long-term Forest Ecosystem Research” of People’s Republic of China (LY/T 1952–2011) [21]. During July 2012, the soil samples were obtained from five different vegetation types: *Quercus acutissima* Carr. and *Robina pseudoacacia* Linn. mixed plantation (QRM), *Pinus thunbergii* Parl. and *Pistacia chinensis* Bunge mixed plantation (PPM), *Pinus thunbergii* Parl. (PTP), *Juglans rigia* Linn. (JRL), and abandoned grassland (ABG). Standard sampling methods were used across vegetation types, namely, at each sample plot of the five different vegetation types, three soil cores (with three replicates collected per core) were randomly collected from a depth of 20 cm and mixed to obtain a composite sample, and a total of 15 soil samples that were collected in July 2012 were then sealed in a hop-pocket. Soil samples were air-dried and passed through a 2-mm screen to remove roots, stones and other debris, and the basic situation of the five vegetation types in the research area is shown in Table 1. Based on the international system of soil size fraction, the soil PSD was described for each sample plot of the five different vegetation types according to the percentages of clay (<0.002 mm), silt (0.002–0.02 mm), and sand (0.02–2 mm) in the sample. The clay, silt and sand fractions were determined with a Laser Particle Size Analyzer (LS13320, Beckman coulter companies, USA) after removing organic matter using the digestion method in a heated hydrogen peroxide solution with sodium hexametaphosphate as a dispersing agent [22]. Part of each air-dried sample was finely ground and passed through a 0.1 mm sieve prior to chemical analysis. Each sample was analyzed in duplicate, and the mean values were calculated.

The soil WRCs of the five vegetation types were measured using a soil WRC tester (H-1400pF, Kokusan, Tokyo, Japan). Three soil samples were randomly collected using the 100 cm^3^ cutting ring at each sample plot of the five different vegetation types. The undisturbed soil in a 100 cm^3^ cutting rings were weighed, allowing it to soak into the water until the soil was saturated, and then, the soil water contents were measured under 1, 10, 30, 80, 100, 300, 600, 800 and 1000 kPa of pressure using the soil WRC tester [23].

**Table 1:** Basic situation of the sampling standards in the study area.

Vegetation Types		Vegetation Coverage (%)	Tree Age (Year)	Elevation (m)	Slope (°)	Aspect ^‡^
Mixed forest	QRM site ^†^	90.3	7	326	10–15	sunny
	PPM site	89.4	7	344	15–25	half-sunny
Pure forest	PTP site	78.8	7	346	15–25	sunny
	JRL site	80.7	6	316	10–15	sunny
Comparison	ABG site	35.2	−	340	15–25	sunny

**^†^** QRM—*Quercus Acutissima* Carr. and *Robina Pseudoacacia* Linn. mixed plantation; PPM—*Pinus thunbergii* Parl. and *Pistacia chinensis* Bunge mixed plantation; PTP—*Pinus thunbergii* Parl.; JRL—*Juglans rigia* Linn.; ABG—Abandoned grassland (the species in the abandoned grassland are *Zoysia japonica* Steud., *Rubia-manjith* Roxb. ex Flem., *Themeda japonica* Tanaka and *Setaria viridis (Linn.)* Beauv., *etc.*). **^‡^** The “Aspect” were divided into three classes at 90° intervals from due north, 0°–45° and 315°–360° were shady slopes, 45°–135° and 225°–315° were half-sunny slopes, and 135°–225° were sunny slopes.

### 2.3. Monofractal Method

When the soil particle size is greater than $R_{i}(R_{i.}>R_{i+1,}i=1,2,3\cdots )$, the cumulative volume of soil particles size *R_i_* and greater $\left(V(r>R_{i})\right)$ () can be expressed as follows [24]:

(1) $$V(r>R_{i})=C_{v}[1-{\left(R_{i}/\lambda_{v}\right)}^{3-D}]$$

where $R_{i}$ is the characteristic scale; $C_{v},\lambda_{v}$ is constant for describing the soil particle shape and scale; and *D* is the monofractal dimension of the soil PSD.

When $R_{i}=0,$ the $V(r>R_{i})$ in Equation (1) becomes the total volume of all soil particles ($V_{T}$), or $V(r>R_{i})=V_{T}=C_{v}$.

When $R_{i}=R_{\max}$, $\lambda_{v}=R_{\max}$ = 2000 μm. Therefore, Equation (2) was derived from Equation (1) as follows:

(2) $$\frac{V(r>R_{i})}{V_{T}}=1-{|\frac{R_{i}}{R_{\max}}|}^{3-D}$$

To calculate *D* from the PSD data, the following logarithmic expression (Equation (3)) was derived from Equation (2) [5]:

(3) $$\log|\frac{V(r<R_{i})}{V_{T}}|=\left(3-D\right)\log|\frac{R_{i}}{R_{\max}}|$$

where the value of *D* is the difference between 3 and the slope of $\frac{V(r<R_{i})}{V_{T}}$ and $\frac{R_{i}}{R_{\max}}$ of the logarithmic linear regression equation; $R_{\max}$ is the maximum soil particle diameter in mm; and the value of $V_{T}$ is the total volume of all soil particles in %.

### 2.4. Multifractal Method

According to the standard particle size division methods for the Laser Particle Size Analyzer, lg(*φ*_i + 1_/*φ*_i_) is a constant across the measurement interval of *I* = (0.02, 2000 μm). To meet the requirements of the multifractal method, *ψ*_i_ = lg(*φ*_i_/*φ*_1_) (with *I* = 1,2,…, 100) was changed. Next, we received a new dimensionless interval of *J* = [0,5], which had 100 equidistant subintervals, *J*_i_ = [*ψ*_i_, *ψ*_i + 1_], *I* = 1, 2 …, 100. In the interval *J*, 2^k^ same-size subintervals were used (ε), ε = 5 × 2^−k^. Every subinterval contained at least one measured value within a k range of 1 to 6. Thus, the multifractal parameters of *D*_0_, *D*_1_ and *D*_1_/*D*_0_ were calculated with Equations (4) and (5) [25,26]. These multifractal parameters varied between −10 and 10, with a step size of 0.5. From these data, we determined the multifractal spectrum of the soil PSD.

(4) $$D(q)\approx \underset{\varepsilon \to 0}{\lim}\frac{1}{q-1}\times \frac{\log\left[\sum_{i=1}^{n(\varepsilon )}u_{i}{\left(\varepsilon \right)}^{q}\right]}{\log\varepsilon }q\neq 1$$

(5) $$D_{1}\approx \frac{\sum_{i=1}^{n(\varepsilon )}u_{i}(\varepsilon )\log u_{i}(\varepsilon )}{\log\varepsilon }q=1$$

where *μ*_i_(*ε*) is the volume percentage of every subinterval; *ε* is the same size subinterval; *q* is the given parameter; and *D*(*q*) is the information entropy. When *q* = 0, *D*(*q*) = *D*_0_, which is the capacity dimension, and measures the span of the soil PSD. When *q* = 1, *D*(*q*) = *D*_1_, *D*_1_ is the information dimension, which provides the irregular degree of the soil PSD. *D*_1_/*D*_0_ (the information dimension/capacity dimension) measures the degree of heterogeneity of the soil PSD.

### 2.5. The Fractal Dimensions of the Soil WRC

We used the soil WRC fractal model devised by Tyler *et al.* [27] to calculate the fractal parameters of the soil WRC using the equation:

(6) $$h=h_{d}{\left[\frac{\theta }{\theta_{s}}\right]}^{1/(D\prime -3)}$$

where the value of *D′* is the fractal dimensions of the soil WRC; *θ* is the soil’s volumetric water content in %; *θ_s_* is the soil saturated water content in %; *h* is the soil matric suction in kPa; and *h_d_* is the soil air-entry suction in kPa. *h* is the vertical axis, and *θ* is the abscissa, and then from these values, the curve was drawn. The power of the fitting curve is determined from $1/(D^{\prime }-3)$, which can be used to calculate the *D′*.

### 2.6. Statistical Data Analyses

A one-way ANOVA (SPSS 17.0) was used to compare the effects of the five vegetation types on soil fractal dimension and other soil properties. The LSD procedure was used to separate the means of these soil properties at alpha levels of 0.05 and 0.01. The results are shown as the mean values ± SE of the three observations for each sample plot.

## 3. Results and Discussions

### 3.1. Soil PSD and Its Fractal Dimension under Different Vegetation Types

**Figure 2:**
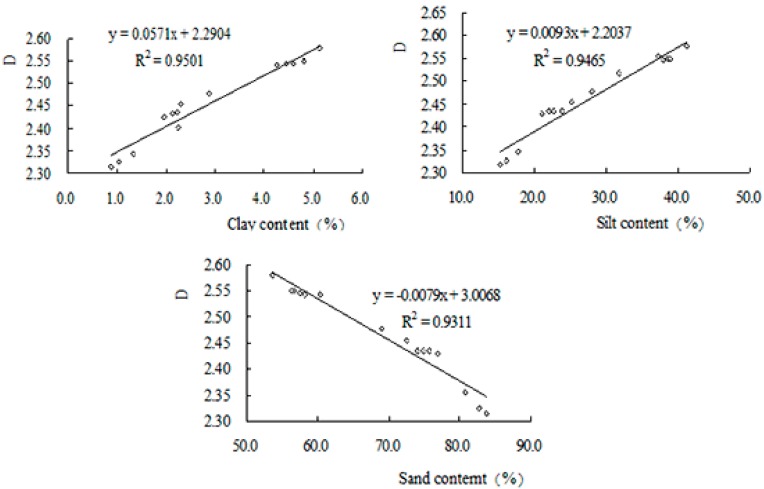
Relationships between *D* and the percentage of different soil PSD. *D*—Monofractal dimension of the soil PSD; PSD—Particle size distribution.

Soil PSD and its fractal dimension under different vegetation types in the study area were calculated (Table 2 and Table 3). The *D* of the soil PSD ranged from 2.3288 to 2.5576, where the maximum *D* was found in QRM vegetation (2.5576), and the minimum *D* was found in ABG vegetation (2.3288). The ANOVA results indicated that *D* significantly differed across vegetation types (*p* < 0.05), where, according to the LSD results, QRM > PPM > PTP > JRL > ABG. The clay content followed the same order across vegetation types, while the sand content followed the reverse order (with ABG having the highest content and QRM having the lowest), suggesting that the *D* of the soil particles was directly proportional to the clay content and inversely proportional to the sand content (Figure 2). This finding demonstrates that the different vegetation measures had obvious effects on improving soil structure, preventing the loss of soil particles (clays and silts) and increasing the values of *D*. Compared to the forest sites (QRM, PPM, PTP and JRL), the soil erosion at ABG sites was more serious and led to the loss of more soil particles (clays and silts), as demonstrated by having the lowest *D* of the different vegetation types.

**Table 2:** Soil particles-size distribution (PSD) under the different vegetation types in the study area.

Vegetation Type	Sand Volume Content (%)					Silt Volume Content (%)	Clay Volume Content (%)
	Very Coarse Sand	Coarse Sand	Sand	Fine Sand	Very Fine Sand		
QRM **^†^**	0.019 ± 0.004 **^a^**	8.54 ± 0.58 ^a,^*	20.61 ± 1.84 **^a^**	18.35 ± 1.07 **^a^**	8.35 ± 0.74 **^a^**	39.06 ± 3.18 **^a^**	4.90 ± 0.51 **^a^**
PPM	0.020 ± 0.005 **^a^**	4.39 ± 0.37 **^b^**	16.43 ± 1.09 **^b^**	25.63 ± 2.16 **^b^**	10.86 ± 0.82 **^b^**	38.02 ± 3.02 **^a^**	4.65 ± 0.48 **^a^**
PTP	5.82 ± 0.52 **^b^**	26.40 ± 2.12 **^c^**	22.16 ± 1.98 **^c^**	12.40 ± 0.96 **^c^**	5.16 ± 0.47 **^c^**	25.68 ± 2.35 **^b^**	2.38 ± 0.32 **^b^**
JRL	8.63 ± 0.65 **^c^**	14.28 ± 1.21 **^d^**	24.11 ± 2.05 **^d^**	20.96 ± 1.78 **^d^**	7.60 ± 0.59 **^d^**	22.00 ± 1.89 **^c^**	2.12 ± 0.25 **^b^**
ABG	8.97 ± 0.62 **^c^**	24.40 ± 2.02 **^e^**	26.95 ± 2.35 **^e^**	18.11 ± 1.02 **^e^**	5.00 ± 0.38 **^e^**	16.40 ± 1.14 **^d^**	1.08 ± 0.12 **^c^**

**^†^** QRM—*Quercus Acutissima* Carr. and *Robina Pseudoacacia* Linn. mixed plantation; PPM—*Pinus thunbergii* Parl. and *Pistacia chinensis* Bunge mixed plantation; PTP—*Pinus thunbergii* Parl.; JRL—*Juglans rigia* Linn.; ABG—abandoned grassland. * Indicates that the columns with different letters are significantly different at *p* < 0.05.

**Table 3:** Soil fractal dimensions under the different vegetation types in the study area.

Vegetation Type	*D* ^†^	*D*_0_	*D*_1_	*D*_1_/*D*_0_	*D′*	*R*^2^
QRM **^†^**	2.5576 ± 0.48 ^a,^*	0.9317 ± 0.09 **^a^**	0.9104 ± 0.07 **^a^**	0.9824 ± 0.10 **^a^**	2.6165 ± 0.50 **^a^**	0.9111
PPM	2.5462 ± 0.43 **^a^**	0.9241 ± 0.09 **^a^**	0.9057 ± 0.06 **^a^**	0.9773 ± 0.10 **^a^**	2.5913 ± 0.47 **^a^**	0.8477
PTP	2.4563 ± 0.37 **^b^**	0.9212 ± 0.08 **^a^**	0.8799 ± 0.05 **^b^**	0.9495 ± 0.09 **^b^**	2.5341 ± 0.42 **^b^**	0.8253
JRL	2.4331 ± 0.35 **^b^**	0.9205 ± 0.08 **^a^**	0.8761 ± 0.05 **^b^**	0.9454 ± 0.09 **^b^**	2.5110 ± 0.40 **^b^**	0.8511
ABG	2.3288 ± 0.0.32 **^c^**	0.9142 ± 0.07 **^b^**	0.8542 ± 0.05 **^c^**	0.9244 ± 0.08 **^c^**	2.4491 ± 0.36 **^c^**	0.8618

**^†^** QRM—*Quercus Acutissima* Carr. and *Robina Pseudoacacia* Linn. mixed plantation; PPM—*Pinus thunbergii* Parl. and *Pistacia chinensis* Bunge mixed plantation; PTP—*Pinus thunbergii* Parl.; JRL—*Juglans rigia* Linn.; ABG—abandoned grassland; *D—*monofractal dimension; *D*_0_—capacity dimension; *D*_1_—information dimension; *D*_1_/*D*_0_—information dimension/capacity dimension; *D′*—is fractal dimensions of soil WRC. * Indicates that the columns with different letters are significantly different at *p* < 0.05.

**Figure 3:**
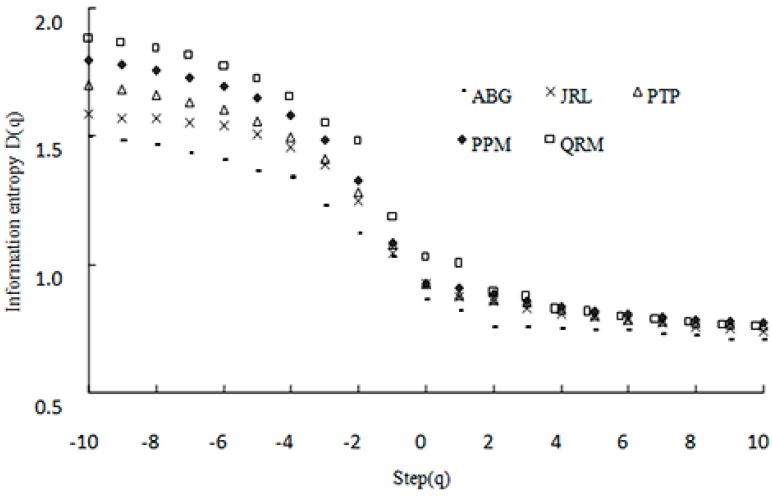
Multifractal spectra of soil PSD under the different vegetation types in the study area QRM—*Quercus Acutissima* Carr. and *Robina Pseudoacacia* Linn. mixed plantation; PPM—*Pinus thunbergii* Parl. and *Pistacia chinensis* Bunge mixed plantation; PTP—*Pinus thunbergii* Parl.; JRL—*Juglans rigia* Linn.; ABG—Abandoned grassland.

By using Equations (4) and (5), the multifractal spectra of soil PSDs in the range of −10 to 10 with a step size of 0.5 under different vegetation types were drawn (Figure 3). Figure 3 showed that the multifractal spectrum of the soil PSD followed a typical reverse S-shaped decreasing function (or curve). *D*_0_ was 0.9142–0.9317 (*n* = 30, *R*^2^ > 0.99), and *D*_1_ was 0.8542–0.9104 (*n* = 30, *R*^2^ > 0.97). All of the multifractal parameters (*D*_0_, *D*_1_) followed a trend of *D*_0_ > *D*_1_.

Table 3 and Figure 3 also showed that the *D*_0_, *D*_1_ and *D*_1_/*D*_0_ values for the five vegetation types followed the same order: QRM > PPM > PTP > JRL > ABG. ANOVA results indicated that the multifractal parameters of woodlands (QRM, PPM, PTP and JRL) were significantly higher than that of ABG (*p* < 0.05). The multifractal parameters of mixed forests (QRM and PPM) were higher than that of pure forests (PTP and JRL) (*p* < 0.05). However, the two types of woodland (QRM and PPM; PTP and JRL) were not significantly different (*p* > 0.05) (Table 4).

**Table 4:** Correlation analysis between soil fractal parameters and soil particles content and soil WRC under the different vegetation types.

Parameter Types	Clay	Slit	Sand	*D* ^†^	*D*_0_	*D*_1_	*D*_1_/*D*_0_
Slit **^†^**	0.935 *						
Sand	−0.950 *	−0.999 **					
*D*	0.944 *	0.957 *	−0.963 **				
*D*_0_	0.663	0.685	−0.687	0.849			
*D*_1_	0.978 **	0.984 **	−0.990 **	0.962 **	0.682		
*D*_1_/*D*_0_	0.969 **	0.971 **	−0.978 **	0.914 *	0.568	0.989 **	
*D*′	0.942 *	0.992 **	−0.993 **	0.986 **	0.769	0.982 **	0.952 *

**^†^** *D—*monofractal dimension; *D*_0_—capacity dimension; *D*_1_—information dimension; *D*_1_/*D*_0_—information dimension/capacity dimension; *D′—*fractal dimensions of soil WRC; * Indicates that the columns are significantly different at *p* < 0.05; ** Indicates that the columns are significantly different at *p* < 0.01.

The value of *D*_0_ describes the span of the soil PSD. Therefore, larger *D*_0_ values indicate a wider soil PSD range, and smaller *D*_0_ values indicate a narrower soil PSD range. The *D*_1_ and *D*_1_/*D*_0_ values reflect the irregularity and the degree of heterogeneity of the soil PSD. Greater *D*_1_ and *D*_1_/*D*_0_ values indicate a greater irregularity and heterogeneity in the soil PSD [25,26,27]. In our study, compared with pure forest land, the mixed forests had a wider range of soil PSDs and had greater irregularity and heterogeneity in soil PSD, while the ABG had the narrowest range of soil PSDs and the least irregularity and heterogeneity in soil PSDs. Across vegetation types, the values of *D*_0_, *D*_1_ and *D*_1_/*D*_0_ were positively correlated with clay and silt contents and negatively correlated with the sand content. These results further corroborate our findings on the ranking order of *D* of soil particles across the different vegetation types. It was potentially explained by the effects of environmental construction projects (such as Return Farmland to Forests Project) on soil structure and function in the study area. Thus, these results indicate that fractal dimensions could adequately describe the influences of different vegetation types on the soil particle composition and the soil PSD. Furthermore, fractal dimensions serve as an important index for soil improvement and development.

### 3.2. Soil WRC and Its Fractal Dimension under Different Vegetation Types

**Figure 4:**
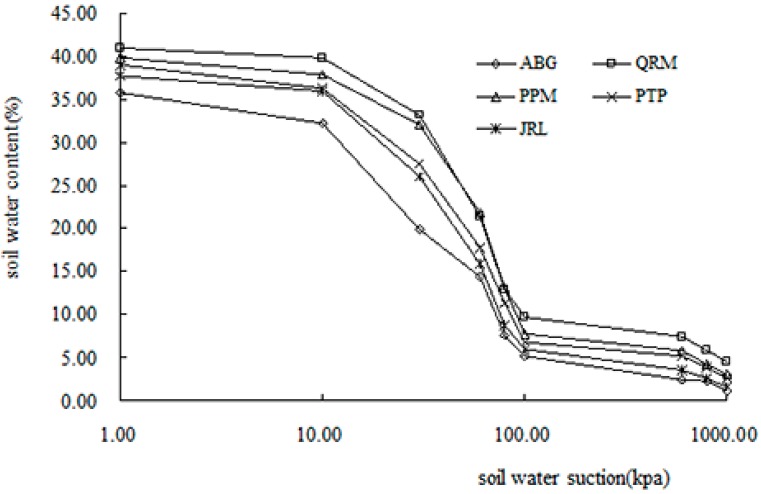
Soil WRC of the different vegetation types in the study area. QRM—*Quercus Acutissima* Carr. and *Robina Pseudoacacia* Linn. mixed plantation; PPM—*Pinus thunbergii* Parl. and *Pistacia chinensis* Bunge mixed plantation; PTP—*Pinus thunbergii* Parl.; JRL—*Juglans rigia* Linn.; ABG—Abandoned grassland.

Soil WRCs of the five vegetation types were measured using the soil WRC tester (Figure 4). The soil WRCs of the five vegetation types were significantly different, and the soil water content, ordered from most to least moisture under the same soil matric suction, was QRM > PPM > PTP > JRL > ABG, with the soil water content of woodlands (QRM, PPM, PTP and JRL) being significantly higher than that of ABG (*p* < 0.05). The fractal dimensions of the soil WRC (*D′*) under the five typical vegetation types were 2.4491–2.6165 and presented a certain regularity (Table 3). The order of *D′* across vegetation types was QRM > PPM > PTP > JRL > ABG, and the clay content followed the same order, while the sand content followed a reverse order. These results showed that the soil water-holding capacity of woodlands (QRM, PPM, PTP and JRL) was significantly greater than that of ABG. This result was potentially explained by the effects of environmental construction projects (such as Return Farmland to Forests Project) on soil structure and function in the study area. Because a high level of soil vegetation coverage of woodlands could intercept rainfall and prevent the rain splash erosion of soil (Table 1), and had strong root systems, they were better ate improving the soil structure than ABG [28]. Thus, the *D′* of woodland were higher than that of ABG, and the soil water-holding capacity of woodlands were also higher than that of ABG.

### 3.3. Correlation Analysis between D′ and D of the Soil PSD

**Figure 5:**
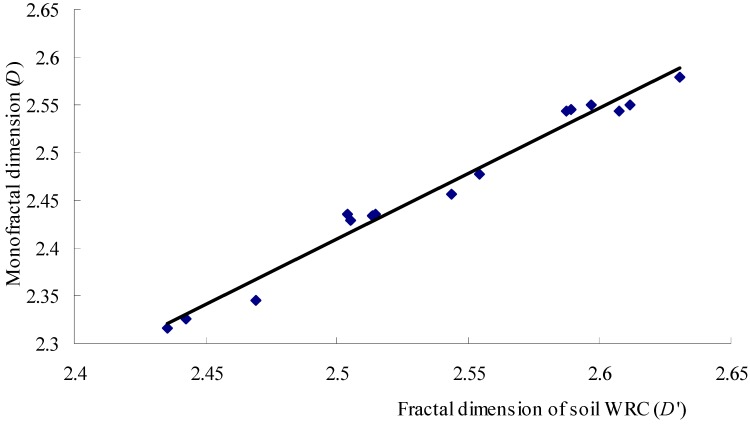
Correlation analysis between *D′* and *D* of soil PSD in the study area.

Soil WRC is an important parameter used to describe soil hydraulic characteristics, which reflects the relationship between the soil water content and soil water matrix suction. Ghanbarian-Alavijeh and Millan [29] found that the *D′* was strongly correlated with water content at 1500 kPa. Because the soil PSD had a significant influence on the soil water matrix suction and the fractal dimension of soil reflected the distribution of soil particles, there was a certain relationship between *D′* and *D* of the soil PSD [30]. In our study, the *D* of the soil PSD was close to *D′* under the different vegetation types (Table 3). The ANOVA results demonstrated a significant positive correlation between *D* and *D′* (*p* < 0.05) (Table 3). Moreover, the *D* of the soil PSD had a linear relationship with *D′* (Figure 5), and a fitting analysis was used to determine the following equation to describe this relationship.

$$D=1.3731D^{'}-1.0239\;(n=15,\;R^{2}=0.9787)$$

Therefore, these results showed that the fractal parameters of the soil WRC and PSD could be used as quantitative indices to reflect the physical properties of the soil. The relationship between the fractal dimension of the soil WRC and PSD were used to describe the corresponding soil WRC.

## 4. Conclusions

Using the monofractal and multifractal theories and methods, the fractal characteristics of soil PSDs and soil WRCs under the five vegetation types were studied in the mountainous land of Northern China. Overall, the following conclusions were drawn.

(1) The fractal parameters of soil PSDs and soil WRCs under different vegetation types were significantly different (*p* < 0.05), and all analyses showed that QRM > PPM > PTP > JRL > ABG. The soil fractal dimensions of mixed forests (QRM and PPM) were higher than that in pure forests (PTP and JRL), and the soil fractal dimensions of woodlands (QRM, PPM, PTP and JRL) were significantly higher than in ABG (*p* < 0.05). These results indicated that the woodland vegetation types had obvious effects on improving the soil structure, preventing the loss of soil, and increasing the soil fractal dimensions. These results also indicated that fractal dimensions could adequately describe the influences of different vegetation types on the soil particle composition and the soil PSD. Furthermore, fractal dimensions served as an important index for soil improvement and development. Therefore, it was necessary to construct different ecological forest types for preventing soil erosion and improving soil structure, especially for the construction and management of mixed forests in a study area.
(2) The *D* of the soil PSD was positively correlated with the fractal dimension of the soil WRC. Therefore, the relationship between the fractal dimension of the soil PSD and WRC could be used to describe the corresponding soil WRC.
(3) The fractal parameters of the soil PSD and WRC were closely related to the soil structure and were used as quantitative indices to reflect changes in the physical properties of the soil. Thus, the fractal parameters of the soil PSD and WRC could also act as an index and theoretical basis for the construction of environmental construction projects (such as Return Farmland to Forests Project) and the evaluation of forest ecological service function in the study area.
